# Extracellular vesicles in *Drosophila* and mammals: Conserved mechanisms and emerging functional roles

**DOI:** 10.70401/EXO.2026.0014

**Published:** 2026-06-23

**Authors:** Kyosuke Yanagawa, Norbert Perrimon

**Affiliations:** 1 Department of Genetics, Harvard Medical School, Boston, MA 02115, USA.; 2 Howard Hughes Medical Institute, Harvard Medical School, Boston, MA 02115, USA.

**Keywords:** Extracellular vesicles, exosomes, *Drosophila melanogaster*, endosome, endosomal sorting complexes required for transport (ESCRT), Rab GTPases, intercellular communication, interorgan communication

## Abstract

Extracellular vesicles (EVs) are membrane-enclosed particles released by cells carrying proteins, lipids, metabolites and nucleic acids that can alter the behavior of recipient cells. In mammalian systems, EVs have been studied extensively as important mediators of intercellular and interorgan communication in development, tissue homeostasis, immunity, regeneration, metabolism, cancer and neurobiology. In parallel, *Drosophila* has emerged as a powerful *in vivo* model for EV research owing to its genetic tractability and the availability of well-established tools for studying interorgan communication. Work in *Drosophila* has shown that EVs participate in synaptic cargo transfer, developmental and reproductive signaling, neuronal homeostasis and systemic immune responses. Importantly, most of the pathways that regulate endosomal sorting, multivesicular body dynamics, membrane budding and vesicle secretion are conserved between flies and mammals. This review summarizes current understanding of EV nomenclature, biogenesis, cargo selection and biological function, with emphasis on points of convergence and divergence between mammalian and *Drosophila* systems. It further discusses the strengths and limitations of *Drosophila* as a model for mammalian EV biology and highlights how comparative approaches can sharpen mechanistic insight and translational EV studies.

## 1. Introduction

Extracellular vesicles (EVs) are heterogeneous membrane-limited particles released into the extracellular space by cells of endosomal or plasma membrane origin that have emerged as a major pathway of cell-cell communication across various biological contexts^[1–3]^. Interest in EVs has expanded rapidly because they can carry membrane proteins, cytosolic proteins, lipids, RNAs, and metabolites, thereby transferring complex molecular information between cells^[2–4]^. In mammals, EVs have been implicated in development, tissue repair, immune regulation, infection, cancer progression, and neurological disease, which are increasingly investigated as liquid-biopsy analytes and therapeutic delivery vehicles^[5–7]^.

At the same time, the field continues to face conceptual and technical challenges. EV preparations are heterogeneous, subtype definitions are not always experimentally established, and the biological significance of detected cargo cannot be assumed without functional validation^[3]^. Accordingly, current consensus guidelines emphasize the precise use of terminology and the application of rigorous characterization approaches to clearly differentiate general claims about EVs from those pertaining to specific subtypes, such as exosomes or microvesicles^[3,8]^. This is particularly important in cross-species studies, in which the strengths of different model systems enable complementary aspects of EV biology to be analyzed. Among model organisms, *Drosophila* provides an excellent model system due to its ease of genetic manipulation and well-established tools for studying interorgan communication^[9–11]^. *Drosophila* combines powerful tissue-specific genetics with a high degree of conservation in membrane trafficking, endosomal sorting, and developmental signaling pathways^[12–14]^. Together, these features make *Drosophila* a valuable comparative model for defining conserved EV mechanisms and their physiological functions across tissues and organisms.

In this review, we discuss current knowledge of EV nomenclature, biogenesis, cargo selection and biological function, with particular emphasis on the conserved and divergent features of mammalian and *Drosophila* EV biology. We compare *Drosophila* and mammalian EV biology with three aims. First, we review conserved mechanisms of cargo selection and vesicle release, with emphasis on the endosomal sorting complexes required for transport (ESCRT) machinery, Rab GTPases, tetraspanins, and lipids. Second, we compare the biological functions of EVs in mammalian physiology and disease with those described in *Drosophila* development and neurobiology. Third, we evaluate the strengths and limitations of *Drosophila* as a mechanistic model for mammalian EV research and translational EV biology. Finally, we highlight the need for further work to define how EV pathways operate across tissues and organisms, and emphasize how comparative studies in mammals and *Drosophila* will advance both mechanistic insight and translational applications.

## 2. Extracellular Vesicle Classification and Characteristics

**Figure 1.**
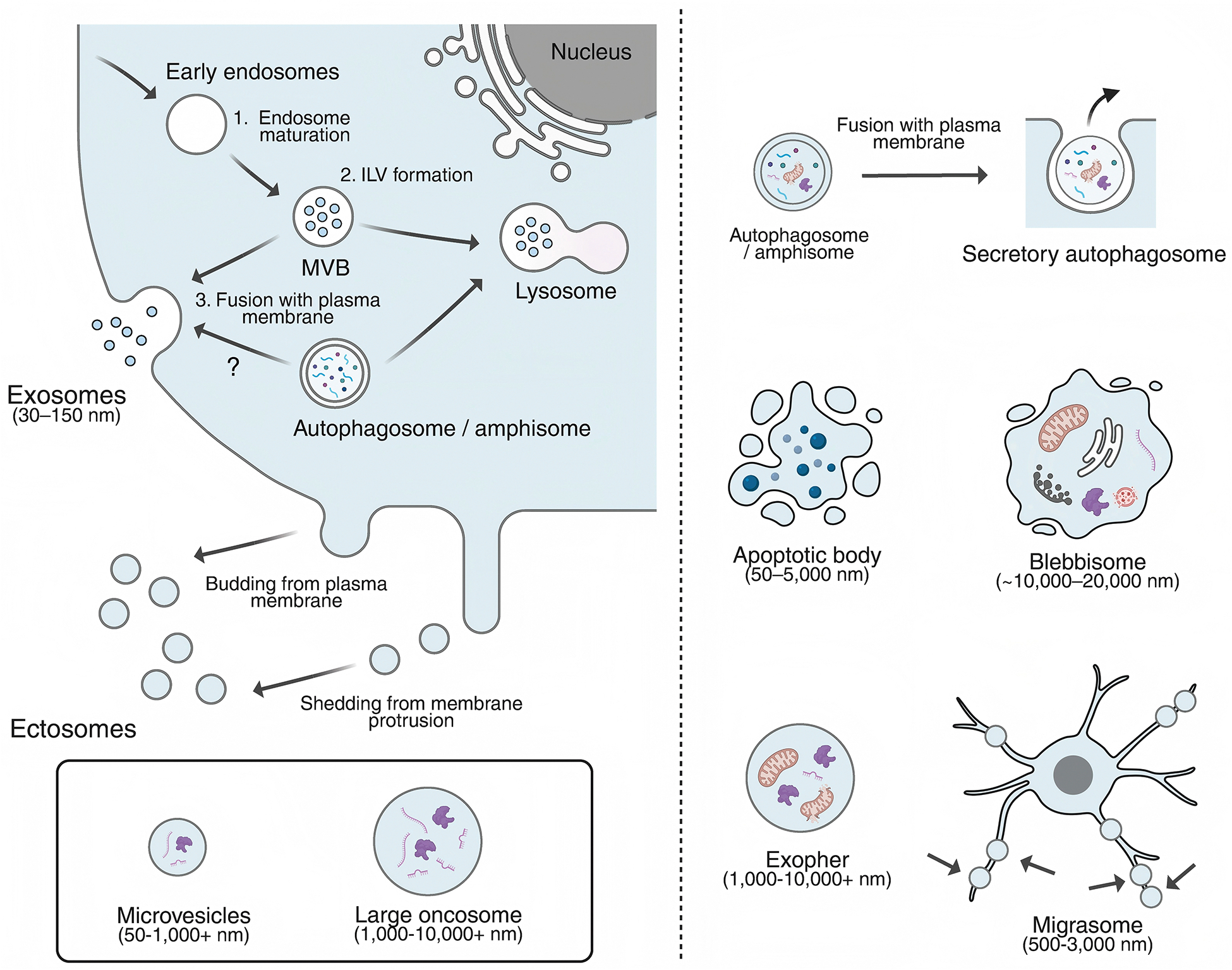
Overview of extracellular vesicle biogenesis and related extracellular structures. Exosomes arise from the endosomal pathway through early endosome maturation, formation of ILVs and MVBs, and fusion of MVBs with the plasma membrane, whereas fusion with lysosomes leads to degradation. Autophagosomes/amphisomes can intersect with this pathway, and secretory autophagosomes provide an additional unconventional route for extracellular release through fusion with the plasma membrane. Ectosomes are generated by direct outward budding from the plasma membrane or membrane protrusions and include microvesicles and large oncosomes. Other extracellular structures shown include apoptotic bodies, blebbisomes, exophers, and migrasomes, which differ in origin, morphology, size, and cargo. Created in BioRender. Yanagawa, K. (2026) https://BioRender.com/ewmsz58. ILVs: intraluminal vesicles; MVBs: multivesicular bodies.

A central issue in EV biology is nomenclature and classification. Historically, terms such as exosomes, microvesicles, microparticles, ectosomes and membrane blebs have often been used inconsistently, sometimes on the basis of size and sometimes on the basis of cellular origin or biogenetic pathway. Current consensus therefore favors the term “EVs” unless the biogenesis pathway has been directly demonstrated^[3,8]^. This recommendation reflects the fact that purification of EVs often contains a heterogeneous fraction of membrane-bound vesicles together with other extracellular particles that overlap in size, density and molecular composition (Table 1)^[2,45–50]^. Exosomes are usually defined as EVs derived from intraluminal vesicles within multivesicular bodies (MVBs) that are released when the MVBs fuse with the plasma membrane (Figure 1)^[2]^. By contrast, microvesicles or ectosomes are generally formed by outward budding and scission of the plasma membrane (Figure 1)^[15]^. Additional membrane-bounded extracellular structures have also been described, including migrasomes, secretory autophagosome- or amphisome-related particles, exophers, apoptotic bodies and, more recently, blebbisomes, further expanding the structural and biogenetic diversity of the EV landscape (Table 1 and Figure 1)^[45,46,51–55]^. Classification of extracellular particles cannot rely on size alone. Commonly used markers, including tetraspanins, ESCRT-associated proteins and flotillins, are useful for enrichment and characterization but are not absolutely subtype specific^[2,3,17]^. General terms such as small EVs or medium/large EVs are therefore often more appropriate than assigning a preparation to a specific subtype when its biogenesis has not been directly shown.

Representative extracellular vesicle and extracellular particle classes. Approximate size ranges and marker profiles can vary across cell types and experimental systems. Exomeres, supermeres, vault particles and lipoproteins are not membrane-bound and therefore are not classified as extracellular vesicles.

EVs are found in many mammalian body fluids, including blood, urine, saliva, breast milk, bronchoalveolar lavage fluid and cerebrospinal fluid^[56,57]^. Their abundance in accessible samples has contributed to the intense interest in EVs as candidate biomarkers^[58]^. Importantly, EVs can carry transmembrane proteins, cytosolic proteins, enzymes, heat-shock proteins, adhesion molecules, RNAs, metabolites and lipids that collectively reflect the state of the donor cell and can influence the phenotype of recipient cells^[3,59]^. In *Drosophila*, EVs have been identified in cultured cells and multiple *in vivo* contexts, including imaginal disc epithelia, neuromuscular junctions, glial cells, and the male accessory gland, supporting that EV-mediated communication is evolutionarily conserved rather than vertebrate-specific^[14,60–68]^.

## 3. Biogenesis of EVs

### 3.1 Exosome biogenesis through the endosomal pathway

Exosome biogenesis is a complex process governed by multiple membrane trafficking pathways that regulate endosomal sorting, membrane remodeling and vesicle trafficking^[1,2]^. The endosomal pathway is central to this process. Early endosomes mature into late endosomes or MVBs, within which inward budding of the endosomal membrane generates intraluminal vesicles, which have two fates: being delivered to lysosomes for degradation or released extracellularly when MVBs fuse with the plasma membrane^[16]^. The ESCRT machinery is one of the best characterized regulators of intraluminal vesicle formation^[69,70]^. ESCRT-0, -I, -II, and -III complexes, together with associated proteins, contribute to cargo clustering, membrane deformation, and membrane scission within endosomes^[69–71]^. However, the contribution of individual ESCRT factors to EV output can vary depending on cell type and vesicle population^[72]^. In HeLa cells and primary dendritic cells, depletion of selected ESCRT components altered exosome secretion and composition in different ways, highlighting the heterogeneity of EV biogenesis^[73]^. These findings underscore that ESCRT dependence differs across EV populations, rather than being a general feature of all EVs^[14]^. Specific autophagy-related factors have also been implicated in exosome biogenesis, further supporting the close relationship between exosome secretion and endolysosomal membrane trafficking^[16]^. In particular, autophagy factors such as ULK1, ATG12-ATG5-ATG16L1 complex, WIPI2/3 or Rubicon can control EV formation independently of canonical autophagy, indicating that pathways classically associated with degradative trafficking also contribute to secretion pathways^[74,75]^. Studies in *Drosophila* neurons revealed that Hsp90 regulates exosome secretion by facilitating MVB fusion with the plasma membrane, a finding supported by cell-free membrane deformation assays. This highlights the utility of the fly model for identifying conserved mechanisms underlying EV biogenesis and release^[76]^. These observations suggest that exosome biogenesis is shaped not only by canonical endosomal sorting machinery but also by broader membrane-remodeling pathways.

### 3.2 Rab GTPases and vesicle trafficking

Rab GTPases constitute another conserved class of EV regulation and are closely linked to the trafficking events that govern vesicle maturation, positioning and release. In mammalian cells, Rab27a and Rab27b have been shown to control steps in the exosome secretion pathway, including docking and spatial positioning of MVBs near the plasma membrane^[77]^. Other Rab proteins, including Rab11, Rab37, Rab35 and Rab39, have also been implicated in recycling-endosome and endosomal trafficking pathways connected to EV release^[73,77,78]^. These findings indicate that EV biogenesis is tightly integrated into general membrane-trafficking networks, with vesicle formation and release depending on pathways that also govern endosomal maturation, recycling and membrane fusion via Rab GTPases.

The EV formation pathway centered on Rab GTPases is strongly conserved in *Drosophila*. Rab11, Syntaxin1A, and Myosin 5 are identified as regulators of EV release from fly synaptic terminals and MVBs containing Evi in presynaptic boutons^[79]^. In *Drosophila* Schneider 2 cells, Rab11 contributes significantly to the production of exosome-like vesicles^[61]^. More recent work has revealed opposing roles for retromer and Rab11 in neuronal EV cargo traffic at presynaptic terminals, with Rab11-positive recycling pathways maintaining EV cargo pools and retromer promoting cargo removal from EV precursor compartments^[80]^. The *Drosophila* male accessory gland further illustrates how Rab-dependent trafficking pathways can generate EVs through tissue-specific endosomal compartments. In accessory gland secondary cells, exosome-like vesicles arise from unconventional Rab11-positive endosomal compartments that are linked to dense-core granule maturation and EV secretion^[63,64,66,81]^. This pathway connects secretory and endosomal trafficking through a Rab6-to-Rab11 transition, showing that accessory gland secondary cells use Rab11-positive endosomes to generate exosome-like vesicles^[64]^. Together, these studies indicate that Rab GTPases provide a conserved trafficking framework for EV formation in flies and mammals, while *Drosophila* further shows that Rab11-dependent pathways can generate EVs through distinct tissue-specific routes.

### 3.3 ESCRT-independent pathways and lipid-dependent membrane remodeling

EV biogenesis can also proceed through ESCRT-independent pathways. In oligodendroglial cells, inhibition of neutral sphingomyelinase reduces EV release, and the released vesicles are enriched in ceramide, supporting a lipid-dependent mechanism of intraluminal vesicle formation^[82]^. Tetraspanin-enriched membrane microdomains also contribute to EV biogenesis and cargo recruitment, and proteins such as CD9, CD63 and CD81 are widely used as EV-associated markers in mammalian systems^[3,15,83]^. Tetraspanins also organize membrane proteins, influence cargo selection and modulate uptake by recipient cells^[84,85]^. Together, these observations indicate that EV biogenesis can be shaped not only by ESCRT-dependent sorting but also by lipid composition and membrane microdomain organization.

The lipid- and microdomain-based pathways are likely relevant in *Drosophila* as well, although the fly literature remains less extensive. Since sphingolipid metabolism, endosomal membrane organization and tetraspanin functions are conserved in flies, *Drosophila* could provide a useful system for studying how membrane composition influences EV budding and cargo sorting^[63,68,86,87]^. A recent study has revealed that microvilli of the wing imaginal disc produce small EVs required for both microvillar integrity and EV biogenesis^[68]^, linking membrane organization to EV formation in epithelial tissue *in vivo*. Together, these observations indicate that ESCRT-independent EV biogenesis can be driven by lipid composition and membrane microdomain organization, highlighting the importance of local membrane architecture in shaping vesicle budding.

### 3.4 Plasma membrane budding and ectosome/microvesicle release

Microvesicles or ectosomes are produced by direct outward budding of the plasma membrane and subsequent scission, highlighting a pathway of EV formation that is mechanistically distinct from endosomal exosome biogenesis^[2,3,88]^. Their production depends on membrane curvature, phospholipid redistribution and remodeling of the actin cortex, often under the control of calcium signals and small GTPases^[3,6,17]^. In addition, recent work has shown that a distinct class of protrusion-derived EVs can arise from plasma membrane protrusions regulated by missing-in-metastasis (MIM), a membrane-deforming protein, and mediate highly efficient protein delivery, further expanding the functional diversity of plasma membrane-derived EV biogenesis^[89,90]^. Compared with exosome biogenesis, mammalian studies of microvesicle production are more extensive than in *Drosophila*, and the fly field still lacks subtype-resolved mechanistic coverage across tissues. Nonetheless, the cytoskeletal and membrane-remodeling machinery needed for ectosome release is conserved, suggesting that further fly studies will be valuable for understanding how direct plasma membrane budding contributes to EV heterogeneity *in vivo*.

### 3.5 Cargo selection and molecular composition

EV cargo is selectively enriched from the donor cell and underlies much of the functional specificity. Common mammalian EV-associated proteins include tetraspanins, flotillins, annexins, heat-shock proteins, ALIX, TSG101, integrins, and cell-type-specific signaling or adhesion molecules^[1–3,17,91]^. Cargo composition is influenced by endosomal sorting, membrane microdomain organization, and donor-cell state, underscoring why EVs can serve both as mediators of intercellular signaling and as candidate biomarkers that reflect pathophysiological change^[7,92]^. EVs can also contain messenger RNAs, microRNAs, long non-coding RNAs, circular RNAs, transfer-RNA fragments, and other small RNAs^[2,74]^. In *Drosophila*, glia-derived EVs provide an *in vivo* example of functional small RNA cargo: glial cells secrete exosomal miR-274 into the hemolymph, where it acts non-cell-autonomously on recipient tissues, including tracheal branches and synaptic boutons^[67]^. The lipid composition of EVs is also functionally important. EV membranes are often enriched in cholesterol, sphingolipids, ceramide-related species, and phospholipids, all of which can influence membrane rigidity, curvature, stability, uptake, and fusion-related properties^[82,91]^. Lipid metabolism can also shape cargo partitioning, as shown by ceramide-dependent exosome biogenesis in oligodendroglial cells^[72,82]^. These findings highlight that EV membranes are active determinants of vesicle identity rather than passive envelopes.

## 4. Biological Functions of EVs in Mammalian Systems

### 4.1 Development, homeostasis, and regeneration

EVs participate in normal mammalian physiology as well as disease, and have been implicated in development, epithelial-stromal communication, tissue repair, stem-cell niche interactions, and homeostatic responses to stress^[2]^. EVs can modulate proliferation, differentiation, migration, and survival of recipient cells by delivering membrane ligands, signaling receptors, enzymes, RNAs, and lipids^[2,92]^. Mesenchymal stromal cell-derived EVs are of particular interest because they have shown regenerative and immunomodulatory potential in a wide range of preclinical injury models^[93]^. Although mechanistic and manufacturing challenges remain, these studies have accelerated interest in EVs as cell-free therapeutic agents.

### 4.2 Immunity and inflammation

Immunity is one of the most intensively studied physiological roles for EV biology. Antigen-presenting cells release EVs that carry major histocompatibility complex (MHC) molecules and other immune regulators capable of shaping adaptive immune responses^[5]^. In addition, EVs participate in inflammation, antimicrobial defense, allergic and autoimmune responses, antitumor immunity, and immunotherapy^[6]^. Because immune-cell-derived EV cargo changes with activation state, EVs are also being explored as biomarkers of inflammatory and infectious disease^[6,94]^. Altogether these studies highlight the dual role of EVs as both carriers of biological information and active mediators of tissue responses.

### 4.3 Systemic metabolism and interorgan communication

EVs are increasingly implicated in systemic metabolic regulation and in communication between distant organs. Beyond local paracrine effects, circulating EVs can act in an endocrine-like manner to transfer regulatory information across tissues, thereby coupling nutritional state, inflammation and tissue function at the organismal level^[95–99]^.

Evidence from mouse models indicates that adipose tissue contributes substantially to the circulating pool of exosomal miRNAs and that adipose-derived EV cargo can regulate gene expression in distant organs, establishing EV-associated small RNAs as an additional class of adipose-derived endocrine signals^[96,100,101]^. The effects of adipose-derived EVs extend across multiple recipient tissues, including liver, skeletal muscle, pancreas, cardiovascular tissues and immune compartments, where they influence insulin sensitivity, lipid handling, inflammation and vascular function^[96,100,102]^. In obesity, these EVs are associated with impaired insulin signaling, hepatic steatosis and vascular dysfunction, whereas EVs derived from healthy adipose tissue, brown adipose tissue or adipose-derived stromal cells instead support tissue repair and metabolic homeostasis^[96,97,100,102–105]^. Together, these findings illustrate how adipose-derived EVs contribute to systemic metabolism and interorgan communication in both physiology and disease.

### 4.4 Cancer progression, metastasis, and biomarker development

Cancer EV biology is among the field’s most mature translational areas. Tumor-derived EVs have been implicated in angiogenesis, stromal remodeling, immune modulation, therapy resistance, and metastatic niche formation^[7,106–109]^. Exosomal integrin repertoires are associated with organotropism metastasis, suggesting that tumor-derived exosomes can help prepare pre-metastatic niches in distant tissues; specifically, exosomal integrins α6β4 and α6β1 were linked to lung tropism, whereas αvβ5 was associated with liver tropism in experimental models^[7]^. These findings establish tumor-derived EVs as active mediators of metastatic conditioning and highlight their potential value as indicators of organotropism spread and disease progression. Since EV cargo can reflect the molecular state of the cell of origin, circulating EVs are also being evaluated as liquid-biopsy analytes. For example, exosomal RNA can improve detection of tumor mutations, and exosome-derived DNA and surface proteins such as PD-L1 may provide complementary information on tumor genotype and immune status^[106,110]^. EV-based liquid biopsy is of considerable interest because EVs are abundant in accessible body fluids, protect their molecular cargo from degradation and carry multiple classes of biomolecules that can report tumor genotype, cellular state and tumor–microenvironment interactions^[58,111,112]^. Overall, these findings position cancer-derived exosomes as both active participants in tumor progression and promising platforms for biomarker development.

### 4.5 Nervous system function and neurodegeneration

In the nervous system, EVs mediate communication among neurons, astrocytes, microglia, oligodendrocytes, and vascular-associated cells. They are implicated in synaptic modulation, stress adaptation, and transfer of lipids and proteins important for neural homeostasis^[15,113–115]^. However, EVs have also been linked to the spread of pathogenic proteins associated with neurodegenerative diseases, including Alzheimer’s disease, Parkinson’s disease, and prion disorders^[113,114,116]^. EVs may contribute both to removal of unwanted biomolecules and to dissemination of disease-associated proteins, while noting that definitive causality in human pathogenesis remains difficult to establish.

## 5. EV Biology in *Drosophila*

### 5.1 Why *Drosophila* matters for EV research

*Drosophila* is especially useful for EV studies because it combines deep conservation of membrane-trafficking pathways with rapid genetics and sophisticated tools for tissue-specific experimental control^[117–119]^. In contrast to many mammalian systems, fly tissues allow direct *in vivo* analysis of vesicle release, donor-cell manipulation, and phenotypic characterization of recipient cells. The larval neuromuscular junction has been influential because presynaptic and postsynaptic compartments are accessible to imaging, genetic perturbation, and ultrastructural analysis. As a result, the fly has become a valuable system for dissecting how endosomal routing, recycling, and vesicle export shape EV-associated signaling and cargo homeostasis.

### 5.2 EV-mediated developmental and reproductive signaling

Studies in *Drosophila* have established important *in vivo* paradigms for EV-mediated signaling and cargo trafficking, which contribute to intercellular communication in multiple *Drosophila* tissues. At the larval neuromuscular junction, Evi/Wntless-containing vesicles function presynaptically in Wingless secretion and postsynaptically in the organization of Wnt-receiving machinery, providing an early model for vesicle-associated communication between neurons and muscles^[120]^. Subsequent work identified Rab11 as a regulator of Evi vesicle release and visualized Evi-containing multivesicular bodies in presynaptic boutons, further supporting an exosome-like trafficking mechanism at synapses^[79]^. In epithelial tissues, EV-associated morphogen transport has also been demonstrated for Hedgehog, which is carried by microvillus-derived vesicles in the wing imaginal disc, demonstrating that EV-associated morphogen transport contributes to epithelial patterning and morphogenesis^[68]^. In the reproductive system, male accessory gland-derived exosome-like vesicles are transferred to females during mating and contribute to post-mating physiological and behavioral responses^[63]^. Together, these examples indicate that fly EVs can regulate developmental and reproductive signaling through communication between distinct cells and tissues.

### 5.3 Neuronal EVs in proteostasis and disease-associated cargo handling

Studies at the *Drosophila* neuromuscular junction have linked neuronal EV pathways to local cargo trafficking, synaptic homeostasis, and proteostasis. Loss of canonical endocytic proteins, including Shibire/dynamin, AP-2, and Nervous wreck, disrupts local EV cargo trafficking at presynaptic terminals and reduces the release-competent EV cargo pool^[121]^. Therefore, synaptic phenotypes in these mutants may arise not only from impaired synaptic vesicle recycling, but also from defective EV cargo handling. In *Drosophila* motor neurons, disruption of ESCRT machinery impairs synaptic EV cargo release but does not abolish signaling by several EV cargoes, including Syt4 and some Evi-dependent activities^[13]^. These findings challenge a simple model in which neuronal EVs primarily act as trans-synaptic signaling vehicles. Instead, they suggest that some proposed EV cargoes may signal through EV-independent and/or ESCRT-independent pathways, whereas ESCRT-dependent EV release can also function in synaptic cargo clearance and proteostasis.

Consistent with a broader role for EV pathways in proteostasis, a *Drosophila* glucocerebrosidase-deficiency model showed altered abundance and turnover of EV-associated proteins. Importantly, genetic reduction of EV production suppressed protein aggregation in this model, suggesting that dysregulated EV metabolism can contribute to pathogenic protein accumulation or spread^[122]^. Although this study does not specifically define these EVs as neuron-derived, it connects EV dysregulation to disease-relevant proteostasis *in vivo*. Together, these studies support a broader model in which neuronal EV pathways regulate synaptic function through cargo trafficking, cargo clearance, and adaptive stress responses, highlighting EVs as multifunctional contributors to synaptic homeostasis rather than simply as trans-synaptic signaling vehicles.

### 5.4 Capsid-associated EV cargo in RNA transfer and neural signaling

Capsid-forming proteins provide a striking example of specialized EV-associated cargo. In *Drosophila*, dArc1 forms capsids that bind RNA and are transported via EVs; among the EV-associated RNAs, dArc1 mRNA has been shown to be delivered to recipient cells^[60]^. At the neuromuscular junction, motor neuron-derived dArc1-containing EVs deliver dArc1 mRNA to postsynaptic muscles^[123]^. Salivary gland-derived dArc1-containing EVs can also enter the hemolymph and reach distant recipient tissues, including tracheal cells, through a targeting mechanism involving the EV-associated surface protein Stranded at second (Sas), which interacts with dArc1 capsids and promotes targeting to Ptp10D-expressing recipient cells^[124^]. Recent work further shows that dArc1 capsid signaling regulates sugar reward valuation in the adult brain: dArc mutants overvalue sugar rewards and show enhanced sucrose responses in γ5 protocerebral anterior medial (PAM) dopaminergic neurons, and this phenotype is rescued by wild-type dArc1, but not by a capsid-deficient dArc1, in serotonergic neurons^[125]^. These findings indicate that dArc1 capsid-associated EV pathways can regulate not only RNA transfer between peripheral tissues, but also neuromodulatory circuits controlling reward valuation. The emerging literature on dArc1, dArc2, and Copia indicates that capsid-forming proteins can define a broader mode of intercellular communication in *Drosophila*^[60,126]^. dArc1 is the best-established example of an EV-associated capsid cargo, whereas Copia illustrates that other retroelement-derived capsids can also participate in cell-to-cell transfer and synaptic plasticity^[126]^. Whether these capsid-forming proteins use shared EV-dependent routes, distinct extracellular pathways, or cargo-specific targeting mechanisms remains an important open question. Together, capsid-forming proteins extend the concept of EV cargo from individual proteins, lipids, and RNAs to higher-order RNA–protein assemblies that can influence synaptic and behavioral physiology.

### 5.5 EVs in immunity and host–pathogen interactions

Beyond developmental signaling and neuronal cargo trafficking, EVs in *Drosophila* also participate in systemic immunity and host–pathogen interactions. Hemocytes secrete exosome-like vesicles containing viral siRNAs that mediate systemic antiviral immunity by delivering antiviral RNAs to distant tissues and promoting protection against infection^[127]^. In contrast, EV-like particles can also be deployed by parasites to manipulate host immunity. Parasitoid wasps inject venosomes into *Drosophila* hosts, where they alter hemocyte behavior and suppress immune responses^[128]^. Together, these findings extend the significance of *Drosophila* EV research beyond developmental and neuronal contexts, highlighting EVs as mediators of both host defense and host–parasite interaction.

## 6. Strengths and Limitations of *Drosophila* as A Model for Mammalian EV Biology

*Drosophila* offers a major advantage for EV research in its causal resolution: genetic manipulation can be restricted to defined cell types and developmental windows, making it easier to distinguish local effects on EV trafficking from broader disruptions in membrane trafficking or endo-lysosomal biology, an important consideration for endosomal regulators such as Rab GTPases and ESCRT components that function in multiple pathways^[12,13,61,65,81]^. The model also enables direct integration of cell biological mechanisms with whole-organism phenotypes through rapid genetics, developmental analysis, and high-resolution imaging in intact animals, providing an efficient platform for identifying conserved EV regulators for subsequent testing in mammalian systems. At the same time, *Drosophila* has clear limitations: it lacks adaptive immunity, does not capture the complexity of mammalian tumor microenvironments or all aspects of circulating EV biology, and has incomplete conservation of some canonical EV markers, so questions related to clinical biomarkers, vascular dissemination, and human tissue-specific pathology still require vertebrate models. More broadly, these limitations intersect with general challenges in the EV field, including vesicle heterogeneity, incomplete recovery by any single isolation method, contamination by non-vesicular particles, and the difficulty of inferring physiological function from cargo detection alone, particularly *in vivo* where membrane debris, alternative secretory routes, compensatory trafficking changes, and endosomal stress responses can mimic EV-specific effects^[3,129,130]^. *Drosophila* should therefore be regarded as a complementary mechanistic model that is especially powerful for pathway dissection, rather than a substitute for mammalian translational systems.

## 7. Conclusion

The next phase of EV research will need to move beyond descriptive analyses towards a more precise mechanistic understanding of vesicle biogenesis, cargo selection, tissue targeting and functional output. Key priorities include resolving EV heterogeneity with greater rigor, defining which cargoes are functionally relevant *in vivo*, and clarifying how EV pathways contribute to systemic physiology and disease across tissues and organs. In mammals, these efforts will be particularly important for understanding metabolic and interorgan communication, including the emerging roles of adipose tissue-derived EVs. At the same time, *Drosophila* should continue to provide a powerful comparative platform for identifying conserved trafficking mechanisms and testing their physiological significance *in vivo*. Continued integration of fly genetics with mammalian cell biology and translational studies should help establish a more predictive and functionally grounded framework for EV biology in health and disease.

## Figures and Tables

**Table 1.** Representative classes of extracellular vesicles and related extracellular particles.

Class	Membrane bounded	Approximate size	Representative markers	Note
Exosomes	Yes	30–150 nm	CD63, CD81, CD9, TSG101, ALIX, flotillin	Defined by endosomal origin[1–3,15–18]
Ectosomes/microvesicles	Yes	50–1,000+ nm	Annexins, ARF6, phosphatidylserine	Generated by outward budding of the plasma membrane[1,2,15,18–20]
Large oncosomes	Yes	1,000–10,000 nm	Variable; tumour-context dependent	Large tumour-associated plasma membrane-derived EVs[3,18,21,22]
Migrasomes	Yes	500–3,000 nm	TSPAN4	Released from retraction fibers during cell migration[23,,51–53]
Secretory autophagosomes/amphisomes	Yes	Not well defined	LC3	Extracellular compartments linked to secretory autophagy[24–27,47,48]
Blebbisomes	Yes	~10,000–20,000 nm	Functional mitochondria, multivesicular endosomes	Exceptionally large EVs blebbed from cancer cells[26,28]
Exophers	Yes	1,000–10,000 nm	Phosphatidylserine, LC3, Tom20	Large extracellular structures implicated in disposal of cellular material[29–31,45,46]
Apoptotic bodies	Yes	50–5,000 nm	Phosphatidylserine	Released from cells undergoing apoptosis; usually considered separately from canonical EVs[3,18,32]
Vault particles/vaults	No	~40–70 nm	MVP, vtRNAs	Non-membranous ribonucleoprotein particles[33–37,50]
Exomeres	No	< 50 nm	No established canonical markers	Non-membranous extracellular nanoparticles[17,38,39]
Supermeres	No	< 50 nm / not fully standardized	AGO2, TGFBI, MET, GPC1, APP, ACE2, PCSK9	Non-membranous extracellular nanoparticles distinct from exomeres and small EVs[40–42,49]
Lipoproteins	No	Variable; overlaps with small EVs	ApoA1, ApoB	Major non-vesicular contaminants in biofluids and EV preparations, especially plasma[43,44]

EVs: extracellular vesicles.
